# Phased Array Ultrasonic Testing of W/EUROFER Functionally Graded Coating

**DOI:** 10.3390/ma18214896

**Published:** 2025-10-26

**Authors:** Ashwini Kumar Mishra, Jarir Aktaa

**Affiliations:** Karlsruhe Institute of Technology (KIT), Institute for Applied Materials, Hermann-von-Helmholtz-Platz 1, 76344 Eggenstein-Leopoldshafen, Germany

**Keywords:** phased array ultrasonic test, non-destructive evaluation, W coating, delamination detection, nuclear fusion

## Abstract

W/EUROFER functionally graded material (FGM) plasma-sprayed coatings are used as a protective layer in nuclear fusion applications. It is vital to develop a non-destructive test method to analyze interface characteristics and detect delamination in coatings. A phased array ultrasonic test method was developed in this work to analyze the coating interface characteristics. Two types of coated samples were tested: first, a W/EUROFER FGM-coated flat small sample, and secondly, a large-scale L-shape 50% W and 50% EUROFER curve-coated sample. The phased array ultrasonic test method reliably detected two separate interfaces in W/EUROFER FGM coating, and no delamination was detected, which was verified by cross-sectional image analysis. Secondly, the phased array ultrasonic test precisely detected delamination created during deposition in a large-scale L-shape 50% W and 50% EUROFER curve coated sample. The accuracy in detecting delamination was verified by cross-sectional images of the interface. The phased array ultrasonic test was found to be a reliable method for detecting delamination in multilayer coatings from small-scale to large-scale curved components.

## 1. Introduction

Tungsten is a promising material for a protective coating over the structural material of the First Wall in various nuclear fusion plants such as the International Thermonuclear Experimental Reactor (ITER) and the DEMOnstration power plant (DEMO) [1]. Different deposition techniques have been developed for W coatings, such as atmospheric plasma spraying [2,3], cold spraying [4,5], vacuum/low-pressure plasma spraying [6,7,8,9,10,11], radio frequency inductively coupled plasma (RF-ICP) [12,13], laser cladding [14,15], physical vapour deposition (PVD) [16,17,18,19], and chemical vapour deposition (CVD) [20,21,22,23]. A W/EUROFER functionally graded coating [6,7,8,9] was developed between the W top coat and the steel substrate to reduce residual stresses resulting from a mismatch between the coefficients of thermal expansion.

These coatings develop residual stress during deposition and thermal stress during fusion operation, since they will be subjected to thermal cycles. These stresses can lead to delamination of the coating from the steel. Hence, it is essential to evaluate the interface characteristics of the W coating over steel.

Various destructive and non-destructive testing (NDT) methods have been developed for evaluating the interface characteristics. Some of the popular destructive testing methods are the four-point bend test, three-point bend test, cantilever beam bending, clamped beam test, modified tension adhesion test (ASTM-C633), indentation test, and shear test [24,25]. The ultrasonic testing [26,27,28,29,30,31] and infrared thermography testing [31,32,33] are commonly used as non-destructive testing methods for evaluating the interface characteristics. Destructive testing quantifies the interface, while non-destructive methods evaluate the interface characteristics qualitatively. It is difficult and time-consuming from a practical perspective to test large-scale components by the destructive testing method, in addition to the fact that it also leads to the destruction of coated components. Hence, a non-destructive method is better for testing interface characteristics for large-scale final components manufactured for an application. The ultrasonic testing method is more effective than infrared thermography [31] for finding interface delamination.

The ultrasonic testing was successfully used for detecting delamination at various coating/substrate interfaces in the literature [26,27,28,29,30,31]. The coating thickness is less than the substrate thickness in most applications. The ultrasonic test was mostly performed in the literature [27,28,29,31] from the back side (substrate side) to create a sufficient gap between the surface echo and interface echo, which helps in the analysis of interface characteristics.

Lian et al. [26] developed an ultrasonic testing method for detecting delamination in the coating/substrate interface. Acrylic plates were used to manufacture a coating/substrate equivalent system, and delamination of more than 1 mm was detected reliably. Afterwards, the ultrasonic testing method successfully detected delamination in ZrO_2_ coating over a steel substrate subjected to thermal shock test. Lescribaa et al. [27] used the ultrasonic test method to investigate delamination at the interface of the plasma spray coating deposited on the substrate with a bond coat in between. Mesrati et al. [28] presented the application of ultrasonic testing in detecting delamination in plasma spray coatings over a substrate subjected to thermal cycles. Kishore et al. [29] investigated delamination in plasma spray coating over a substrate with a bond coat during thermal ageing, which develops thermally grown oxides (TGO). An effect of TGOs on the reflected echo was studied with thermal ageing.

Rachidi et al. [31] performed phased array ultrasonic testing of plasma-sprayed Ni-WC coating over steel substrate with a varying number of excited elements, coating thickness, and focusing depth. The optimized parameters were used to investigate an artificial defect in the coating. A defect of 1 mm in diameter was successfully detected by the method.

Chen et al. [30] performed an ultrasonic test on plasma spray coating over a substrate with a bond coat from the front coating side. Although delamination at the coating interface was detected, the surface and interface echoes were very close, which makes the analysis difficult, as expected. A similar observation was found by Grammes et al. [9] while performing the test from the front coating side. An interface echo could not be distinguished from the surface echo.

Taheri et al. [34] reported that phased array ultrasonic testing is better than conventional ultrasonic testing for defect detection in composite materials. Phased array ultrasonic testing has an improved signal-to-noise ratio (SNR) and signal attenuation as compared to conventional ultrasonic testing, which makes defect detection better. Based on the comparison available in the literature, phased array ultrasonic testing was used in this work.

The above-described literature demonstrates that ultrasonic tests detect delamination reliably in simple two-layer to complex multi-layer plasma spray coating systems. But a detailed methodology explaining the correlation between A-scan results and a C-scan is not available. A separate C-scan analysis for interface and backwall (coating outer surface) echoes was not performed.

The objective of this work is to develop a detailed methodology for detecting delamination by phased array ultrasonic testing (PAUT) of W/EUROFER functionally graded material (FGM) coating over a steel substrate. The work was carried out in two steps. In the first step, a PAUT test was performed on a W/EUROFER FGM-coated flat sample, and the results were verified using cross-sectional image analysis. A correlation between A-scan, B-scan, and C-scan of the interface and backwall echo was explained. In the second step, a PAUT test was performed on a large-scale L-shape 50% W and 50% EUROFER curve coated sample. A C-scan image was generated with the test, which detects the delamination in the coating. The cross-sectional image analysis of detected delamination sites verified the testing and analysis methodology.

## 2. Materials and Methods

### 2.1. Materials

**Figure 1:**
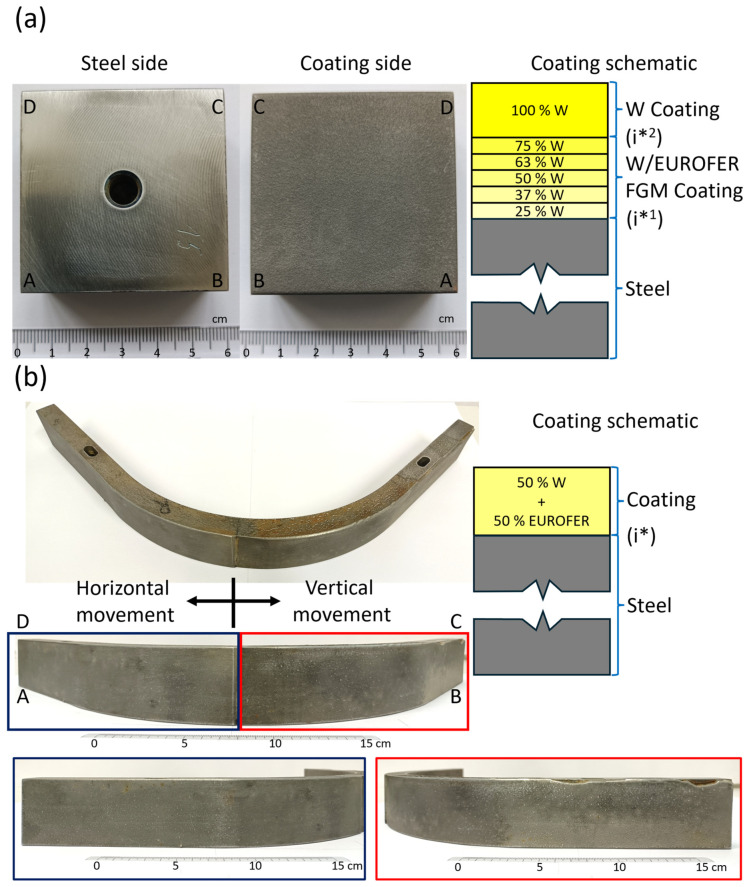
(**a**) W/EUROFER FGM coated flat sample with schematic of coating cross-section. The vertices of the sample are marked as A, B, C, and D (**b**) L-shape 50% W and 50% EUROFER curve coated sample with schematic of coating cross-section. The vertices of the sample are marked as A, B, C, and D.

Two coated samples were tested in this work. First was a W/EUROFER functionally graded material (FGM) coated flat sample deposited by low-pressure plasma-spraying (LPPS) on P92 steel substrate of dimension 48.2 × 48.1 × 20 mm^3^, as shown in Figure 1a. These coatings were deposited by COATEC GmbH (Schlüchtern, Germany), and the details of the deposition parameters are given in reference [9]. The coating consists of a top W coating of thickness 0.88 ± 0.01 mm and a W/EUROFER FGM coating of thickness 1.31 ± 0.01 mm between the top W coating and steel, as shown in Figure 1a. The FGM coating consists of 5 interlayers with varying volume fractions of W from 25% to 75% from steel to the top W coatings. There were mainly 2 significant interfaces in the coated system. Interface 1 (i*^1^) was between the W/EUROFER FGM coating and steel, and interface 2 (i*^2^) was between the W coating and W/EUROFER FGM coating. A cross-sectional sample was cut by an electrical discharge machining (EDM), followed by standard metallography polishing. A cross-sectional image of the coating to observe the interface was taken by scanning electron microscopy (EVO MA10, Zeiss, Oberkochen, Germany).

The second sample was vacuum plasma sprayed 50% W and 50% EUROFER coating deposited on an L-shaped, curved P92 steel substrate of 20 mm thickness and 50 mm width. The substrate has two 150 mm long flat sections, with a quarter curve section having an outer radius of 200 mm, as shown in Figure 1b. The coating was deposited at Forschungszentrum Jülich, Germany, using the vacuum plasma spray (VPS) method, and the details of deposition are given in reference [35]. Half of the L-shaped part was coated by horizontal movement, and the other half was coated by vertical movement of the plasma spray gun. The coating was more than 1 mm on both sides. There was one interface (i*) between coating and steel substrate. A visible delamination was observed on the right side, where the coating was deposited by vertical movement. Since these coatings had delamination, larger pieces of the samples were prepared for cross-sectional image analysis (nearly 150 mm long) to avoid complete failure of the coating during sample cutting. A cross-sectional image was taken by an optical microscope (VHX-1000 digital microscope, Keyence, Osaka, Japan) to observe the interface.

### 2.2. Phased Array Ultrasonic Testing

**Figure 2:**
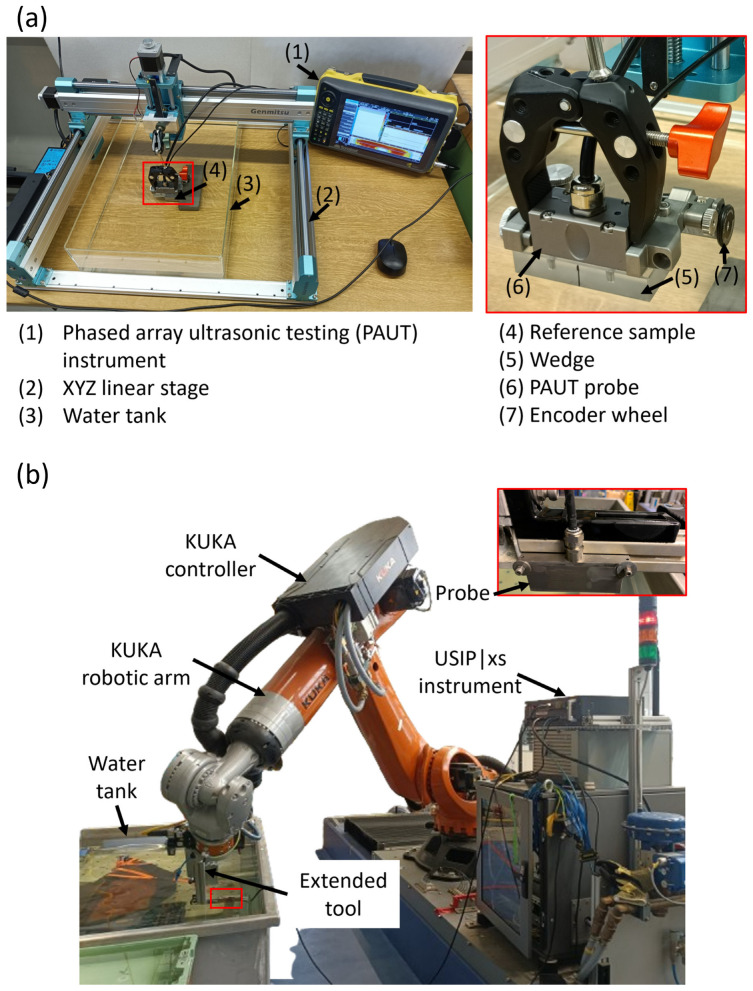
(**a**) Phased array testing setup (Sonatest VEO system) used for testing W/EUROFER FGM-coated flat sample, and (**b**) Krautkrämer HydraStar robotic ultrasonic system used for testing L-shape 50% W and 50% EUROFER curve coated sample. The inset red box shows the magnified view of the probe.

Phased array ultrasonic testing (PAUT) was performed on a W/EUROFER FGM-coated flat sample by the Sonatest VEO system using a phased array probe (10 MHz, 64-phased array elements). The testing setup is shown in Figure 2a. The phased array probe was attached to a polymer wedge, which acts as a delay block. An encoder with an encoder wheel was connected to the probe, which rotates with the probe’s movement and records the linear movement of the probe. The probe was connected to an XYZ linear stage for moving the probe over the sample. The probe and sample were immersed in a water tank, as water acts as a coupling medium between the wedge and the sample surface. A width of 29.4 mm was covered in 1 raster movement of the probe. Hence, a total of two raster scans were performed for scanning the complete W/EUROFER FGM-coated flat sample. The PAUT test was performed on the back side of the sample. The wedge was in contact with P92 steel. So, an ultrasound velocity of 5910 m/s corresponding to the sound velocity of P92 steel [36] was used for testing. Data from A-scan, B-scan, and C-scan were recorded during the test. The initial data was analyzed using UT Studio Standard 3.19.2 software and further plotted using the OriginPro 2023 software.

Phased array ultrasonic testing of an L-shape 50% W and 50% EUROFER curve-coated sample was performed using the Krautkrämer HydraStar (Waygate Technologies, a Baker Hughes company, Hürth, Germany) robotic ultrasonic system, as shown in Figure 2b. A USIP|xs instrument with LINA (10 MHz, 128 elements) phased array probe was used in the testing system. A phased array probe was connected to an extended tool of the Kuka robotic arm, and the tool movement was recorded in the controller. Such robotic systems are useful for testing industrial-scale components having complicated shapes. The test was conducted at the company Baker Hughes Digital Solutions GmbH (Hürth, Germany).

The test was performed in a water immersion tank from the back substrate side with a 40 mm distance between the probe and substrate. The distance between probe and substrate was used as a water delay block. The test was performed using a sound velocity of 5910 m/s, which is the same as the previous sample. This testing system records the C-scan data based on the reference A-scan.

## 3. Results and Discussion

**Figure 3:**
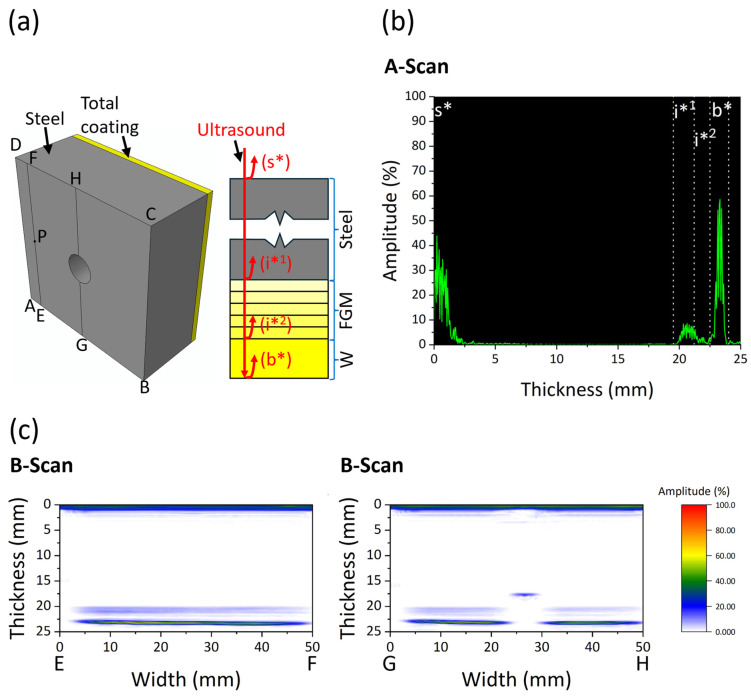
(**a**) Schematic of the W/EUROFER FGM-coated flat sample with ultrasound signal. The vertices of the sample are marked as A, B, C, and D. Points E, F, G, H, and P are marked as reference points for further analysis (**b**) A-scan of point P (**c**) B-scan image along EF and GH of the sample.

Figure 3a shows the schematic of the W/EUROFER FGM-coated flat sample with an ultrasound signal. Figure 3b shows the A-scan at point P of the sample. A-scan data were collected during the phased array ultrasonic testing of the sample. A-scan data show that four echoes were observed during the testing. The first echo was a surface echo (s*) appearing from the surface of the substrate. Afterwards, the second and third echoes were observed from two different interfaces. The interface echo (i*^1^) was observed from the FGM coating/steel substrate interface, and the interface echo (i*^2^) was from the W coating/FGM coating interface. The fourth echo was the backwall echo (b*) from the surface of the top W coating.

It was observed that the interface echo amplitude was much lower than the backwall echo. This is because of the strong bonding at the interface. In case of interface delamination, a higher amplitude (strong) interface echo and a lower amplitude (weaker) backwall echo will be obtained. Figure 3c shows the B-scan image along EF and GH of the sample (Figure 3a). A B-scan image demonstrates the cross-sectional view of the sample, where the x-axis represents the dimension along the sample width and the y-axis represents the thickness of the sample. We can observe the four echoes in the B-scan of the sample. The colour map indicates the amplitude of the echoes. The red colour represents the highest amplitude, indicating a strong echo, while the light blue colour represents the lowest amplitude, which means a weaker echo.

The interface echo is weaker along EF and GH, with a strong backwall echo, indicating a strong interface bonding along these cross-sections. There was a hole in the sample along GH, which was also visible in the B-scan.

**Figure 4:**
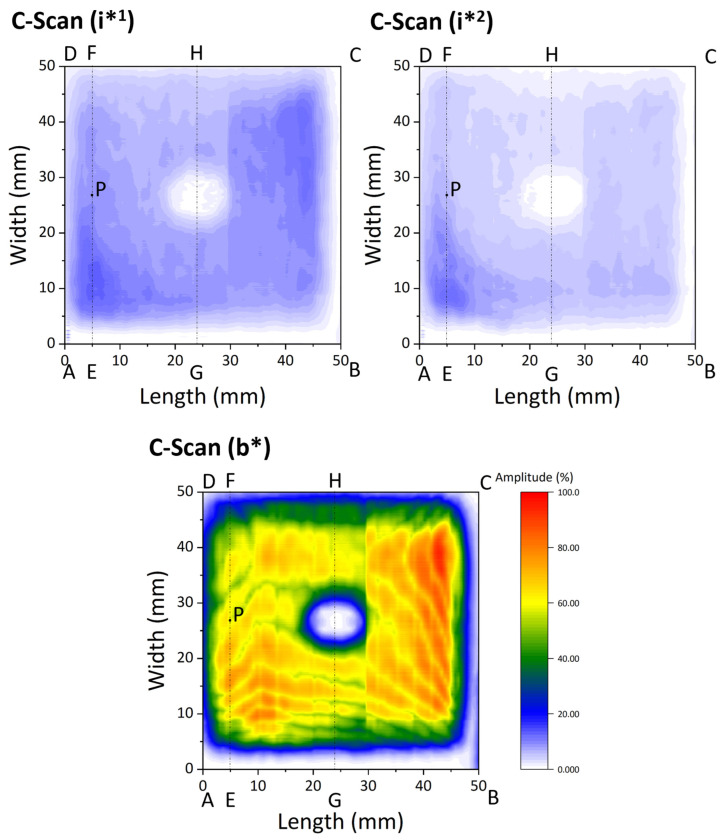
C-scan image of W/EUROFER FGM-coated flat sample.

Figure 4 shows the C-scan image of the W/EUROFER FGM-coated flat sample. Three C-scans were obtained for i*^1^, i*^2^, and b*. These C-scans were plotted based on the gate selected using the A-scan, as shown in Figure 3b. Gate values are the depth range from which maximum amplitudes are determined and plotted. The C-scan (i*^1^) shows the maximum amplitude of echo in the depth range of the interface between the W/EUROFER FGM coating and steel substrate. Similarly, the C-scan (i*^2^) and C-scan (b*) show the maximum amplitude of echo in the depth range of the interface between the W coating and W/EUROFER FGM coating, and W-coating top surface, respectively. The C-scan represents a maximum amplitude of ultrasonic echo for a particular gate (depth range), helping in the analysis of a large area. A colour map was used, similar to the B-scan. C-scan (i*^1^) and C-scan (i*^2^) images are mostly light blue in colour, which indicates weaker echoes and a strong interface bonding without defects. The C-scan (b*) image is mostly red, yellow, and green in colour, corresponding to a stronger backwall echo and strong interface bonding. Lines EF and GH are marked in the C-scan data. A correlation between the B-scan and C-scan can be observed by comparing Figure 3c and Figure 4. A weaker echo was observed for i*^1^ and i*^2^ in the B-scan, which is also consistent in the C-scan image. Similarly, a strong amplitude for b* was consistent in the B-scan and C-scan.

**Figure 5:**
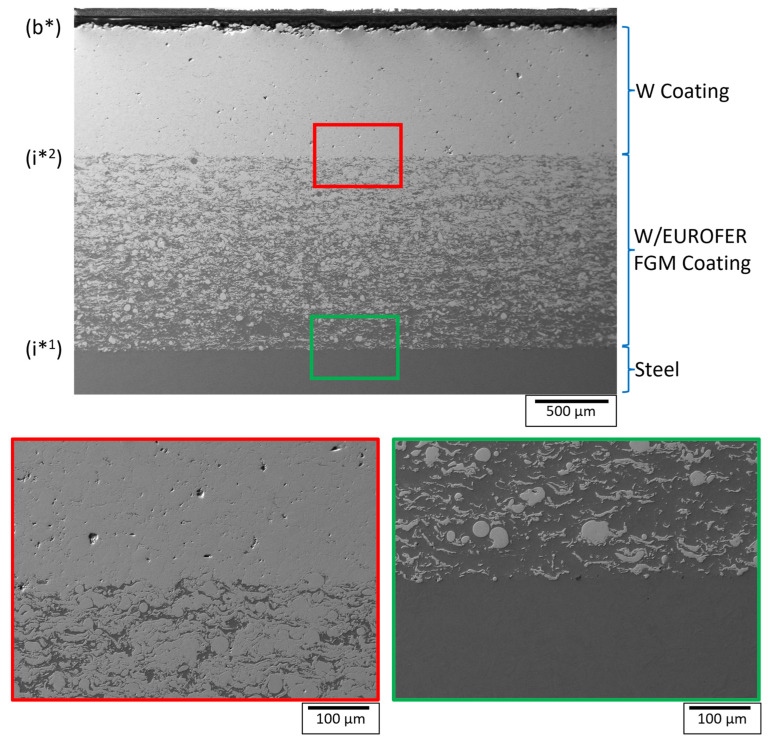
Cross-sectional image of W/EUROFER FGM coating near point P.

Figure 5 shows the cross-sectional image of the W/EUROFER FGM coating near point P. The cross-sectional image was taken near point P to verify the ultrasonic test results. The cross-sectional image shows a W coating on the top and a W/EUROFER FGM coating between the W coating and the steel substrate. The interfaces (i*^1^) and (i*^2^) are indicated in the image with the backwall (b*). The interface between the W coating and W/EUROFER FGM coating was found to be bonded well without any cracks. A magnified image of this interface was shown in Figure 5 (red outline).

Similarly, the interface between the W/EUROFER FGM coating and steel substrate was without any cracks and qualitatively a strong interface. A magnified image of this interface (Figure 5, with the green outline) confirms a strong bonding.

This proves the reliability of ultrasonic test results. A weaker interface echo and a strong back wall echo indicate a strong bonding at the interface, which was confirmed by the cross-section image analysis. This validates the methodology for ultrasonic testing.

**Figure 6:**
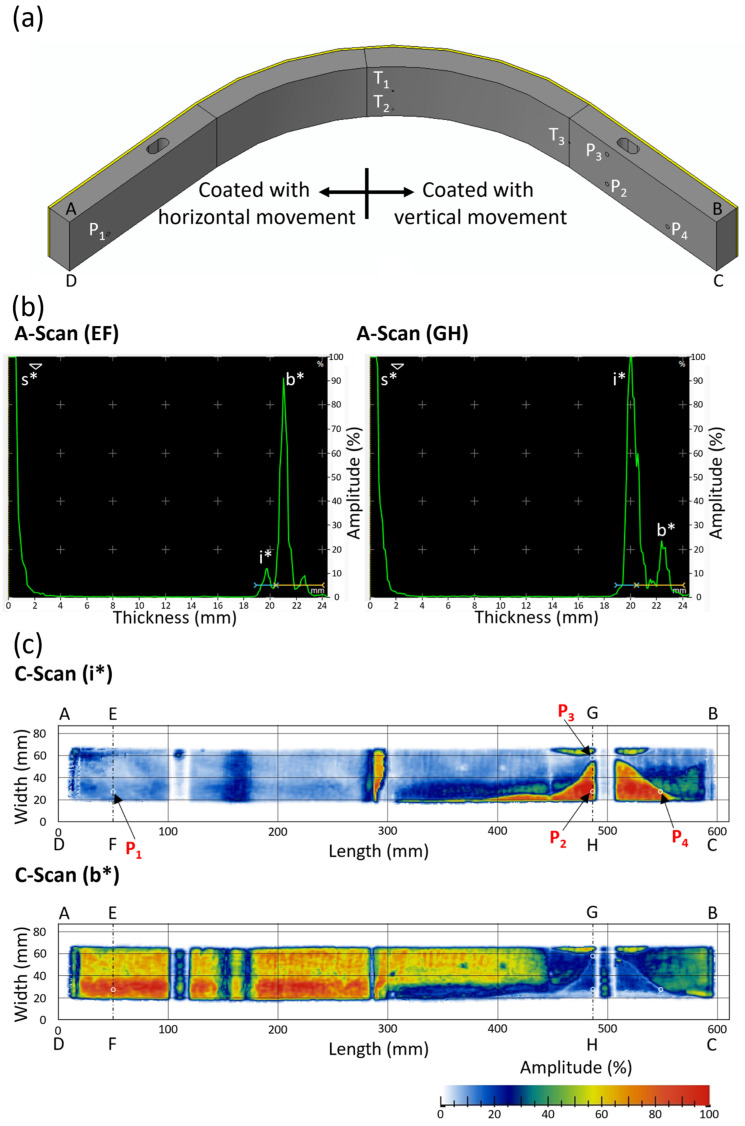
(**a**) Schematic diagram, (**b**) A-scan, and (**c**) C-scan of L-shape 50% W and 50% EUROFER curve coated sample. The vertices of the sample are marked as A, B, C, and D.

Figure 6 shows the phased array ultrasonic test results for the L-shape 50% W and 50% EUROFER curve coated sample. Figure 6b shows the A-scan echo max along the width at two different positions (EF and GH) of the sample. EF and GH are also marked in the C-scan image. An A-scan echo max shows an envelope signal along a width, including the maximum echo from each A-scan along the width. The A-scan echo max was used for testing large components since it is not possible to analyze individual A-scans. Three echoes were observed in the A-scan: surface echo (s*), interface echo (i*), and backwall echo (b*). There was only one interface in this sample between the coating and the steel substrate.

The A-scan along EF shows the A-scan from the coating, which was deposited using horizontal movement. In this case, the interface echo was weak (low amplitude), and the back wall echo was strong (high amplitude). This indicates good bonding between the coating and steel in that region. The A-scan along GH shows the A-scan from the coating, which was deposited by vertical movement. In this case, the interface echo was found to be strong while the back wall echo was weak. This indicates the possibility of delamination along GH. The C-scan images were plotted in Figure 6c for the interface echo and backwall echo. The gate values (depth range) for the C-scan interface and backwall were shown in the A-scan plot (Figure 6b).

The left sides of the C-scan (i*) and C-scan (b*) images (Figure 6c) shows the C-scans of the interface and backwall for coatings that were deposited by horizontal movement, respectively. Like the previous test, here, also light blue colour indicates a low amplitude (weak) echo, while green, yellow, and red colours indicate a high amplitude (strong) echo, respectively. A weak interface echo (light blue colour region) was found on the left side of the C-scan (i*) image, while a strong backwall echo (green, yellow, and red colour region) was found on the left side of the C-scan (b*) image. A weak interface echo and strong backwall echo indicate good interface bonding with no delamination in coatings deposited by horizontal movement.

The right sides of the C-scan (i*) and C-scan (b*) images (Figure 6c) shows the C-scans of the interface and backwall for coatings deposited by vertical movement, respectively. The right sides of C-scan (i*) and C-scan (b*) were found to be a mixed region of weak and strong echoes. The region with strong interface echo (green, yellow, and red colours) on the right side of the C-scan (i*) image reveals delamination at the interface. This can be correlated with the region with a weak backwall echo (light blue) on the right side of the C-scan (b*) image. The strong interface echo region on the right side of the C-scan (i*) image corresponds to a weak backwall echo region. This indicates the presence of delamination in that region.

At the centre of the sample, a strong interface echo was observed (C-scan (i*)). This was due to a change in the thickness of the coating, as shown in Figure 1b. Three holes (marked as T_1_, T_2_, and T_3_ in Figure 6a) of diameter 1.5 mm and 18 mm deep were visible in the C-scan (Figure 6c). These holes were machined for the thermocouples to measure temperature during deposition. The area just below the bore cannot be analyzed because the interface and backwall echoes get masked in this region.

**Figure 7:**
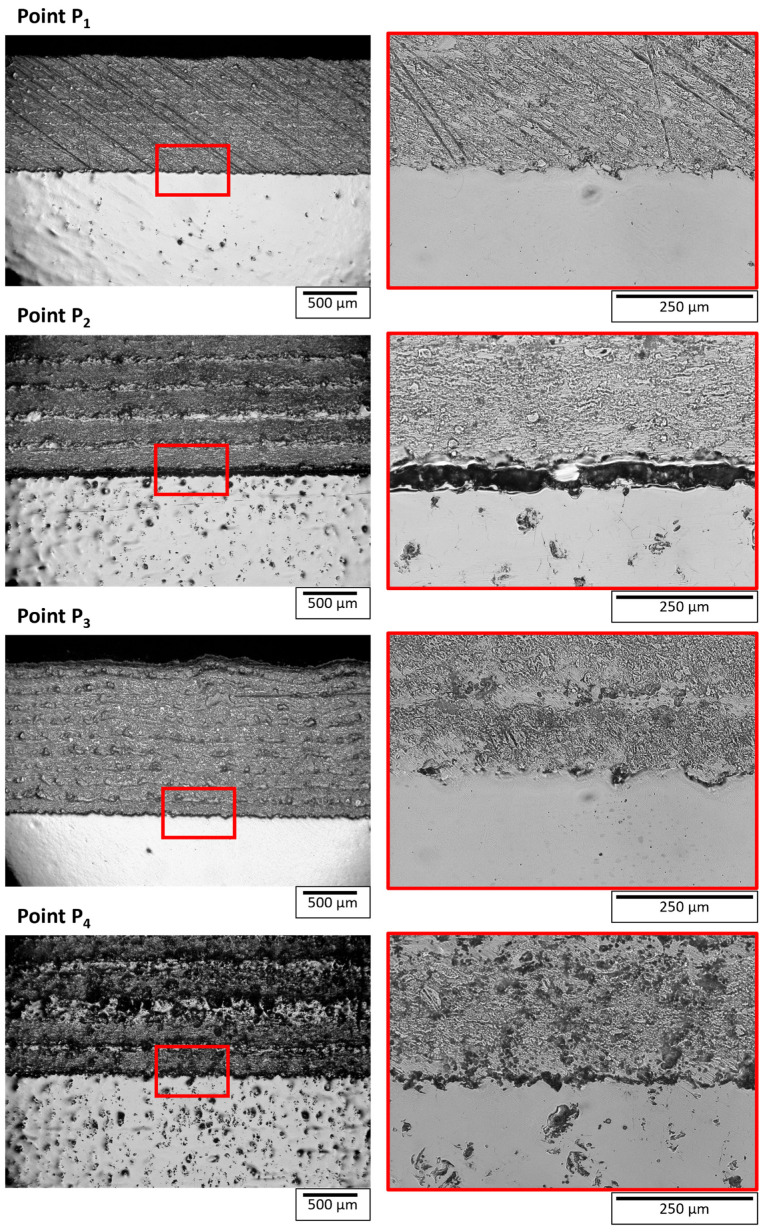
Cross-sectional image of L-shape 50% W and 50% EUROFER curve coated sample near points P_1_, P_2_, P_3_, and P_4_. The red box indicates the area shown in the higher magnification on the right side.

A cross-sectional image analysis of the coating/steel interface was performed to verify the phased array ultrasonic test outcomes. Figure 7 shows the optical microscope image of the coating/steel cross-section near points P_1_, P_2_, P_3_, and P_4_ corresponding to the C-scan images shown in Figure 6c. All the optical images have some marks and pits formed during the grinding and polishing process. These samples had a possible delamination, so a large size of samples were cut to avoid complete failure of the coating from the delamination site. Due to the larger size of the samples, they were polished without mounting, which resulted in some polishing artifacts. Nevertheless, the objective was to observe the interface, which was fulfilled by these images. Point P_1_ was from the region of the coating deposited by horizontal movement. No crack or delamination was detected at the interface of coating/substrate near this point, which was consistent with the results of phased array ultrasonic testing.

Points P_2_, P_3_, and P_4_ were from the region of the coating deposited by vertical movement. A delamination was detected between the coating and steel near points P_2_ and P_4_. Points P_2_ and P_4_ correspond to the region with strong interface echo and weak back wall echo in C-scan (i*) and C-scan (b*), respectively, as shown in Figure 6c. The phased array ultrasonic result (C-scan) indicated the delamination at these points, which is now validated by the cross-section images. Point P_2_ lies between a larger delaminated region, while point P_4_ lies at the corner of the delaminated region, as indicated by the C-scan image (Figure 6c). The effect of the point’s position can be correlated with the delamination opening (distance between coating and steel). The delamination opening near point P_2_ was larger than near point P_4_. In case of delamination, a coating/air interface is created, which gives a higher amplitude of echo even for a small delamination opening, which was detected in C-scan (i*) as shown in Figure 6c (point P_4_).

No delamination was found at point P_3_, as shown in Figure 7. Point P_3_ lies in the region of weak interface echo and relatively strong backwall echo, as shown in Figure 6c. The C-scan results indicate a bonding without delamination between coating and steel, which was confirmed by the cross-section image at point P_3_.

Based on the analysis of vertical and horizontal movements of deposition, the horizontal movement of deposition leads to a more homogeneous temperature distribution. Details regarding the reason for delamination were presented in reference [35] and are not discussed here since the objective of this work is to develop phased array ultrasonic testing for detecting delamination in coated components.

A good correlation was found between phased array ultrasonic test results and cross-section images. This validates the application of phased array ultrasonic testing for detecting delamination in small to large-sized coated components.

It is important to obtain a distinguishable interface echo and backwall echo for the reliable detection of delamination. In this work, we obtained interface echoes in both samples, and a C-scan was created by defining a gate (depth range). If the coating thickness is very low, the interface echo becomes superimposed with the backwall, and distinguishable gates cannot be defined. A similar superimposition of the surface echo and interface echo occurs when the tests are performed from the front side. Grammes et al. [9] showed the ultrasonic test results with the test performed from the front side, which led to the superposition of the surface echo and interface echo. A reliable detection of delamination is not possible due to such superposition. The minimum coating thickness that can be tested depends on the axial resolution, which can be calculated by an analytical formula (wavelength/2) [37] considering a single wave of an ultrasound pulse. Considering the ultrasound frequency of 10 MHz and velocity of 5910 m/s used in this work, the theoretical minimum coating thickness (axial resolution) is 0.3 mm. In this work, a minimum thickness of 0.88 mm W coating was tested, which created a distinguishable interface echo, as shown in Figure 3b. W/EUROFER FGM coating consists of five interlayers, but separate echoes were not found between them. There are two possible reasons: first, the material properties are very similar for these consecutive interlayers, and second, the thickness of each interlayer is less than the axial resolution.

Artificial delaminated cracks were used in the study of ultrasonic testing in the literature [26,31]. In this work, a naturally delaminated coating was used for testing the reliability of the phased array ultrasonic test. Since the delamination was natural, a controlled crack length was not created. Hence, a minimum crack length could not be measured. The minimum delaminated crack length of 1 mm can be measured by ultrasonic testing, as recommended in the literature [26,31].

A phased array ultrasonic testing of large-scale plasma-sprayed coatings/substrate interface using a combination of A-scan, B-scan, and C-scan, along with validation using cross-sectional image analysis, is the novelty of this work. The application of PAUT in coating was limited to small-scale testing and using an artificial crack in the literature [31]. A proper correlation between A-scan, B-scan, and C-scan was not available. This work describes the methodology and verifies it in detail, which will be useful for testing future coated components of the First Wall of nuclear fusion plants and other relevant applications.

## 4. Conclusions

In this work, a reliable phased array ultrasonic test method was described for plasma-sprayed coating samples to investigate the interface characteristics. The main conclusions of the present work are given below:

1. A detailed analysis was provided for the ultrasonic echo in W/EUROFER FGM coating over a steel substrate using the A-scan, B-scan, and C-scan. Distinguishable echoes were obtained from two different interfaces and the backwall in the coating, which were used to create a C-scan for the interfaces and backwall. No delamination was detected at any interface in the coated sample.
2. The reliability of the phased array ultrasonic test was confirmed by image analysis of the cross-section.
3. A C-scan was obtained for the interface and backwall in testing the L-shape 50% W and 50% EUROFER curve coated sample. The C-scan detected the position of the delaminated area, and a good correlation was achieved between the C-scan of the interface and the backwall. The accuracy of the phased array ultrasonic test in detecting delamination was confirmed by cross-sectional image analysis of the coating/substrate interface.
4. A successful testing of an L-shape 50% W and 50% EUROFER curve coated sample shows that the phased array ultrasonic testing method is a reliable NDT method for detecting delamination in large-scale coated components for future fusion applications.

## Data Availability

The original contributions presented in this study are included in the article. Further inquiries can be directed to the corresponding author.

## Abbreviations

Abbreviations used in this manuscript are given below:

FGM	Functionally graded material
ITER	International Thermonuclear Experimental Reactor
DEMO	DEMOnstration
RF-ICP	Radio frequency inductively coupled plasma
PVD	Physical vapour deposition
CVD	Chemical vapour deposition
NDT	Non-destructive testing
TGO	Thermally grown oxides
SNR	Signal-to-noise ratio
PAUT	Phased array ultrasonic testing
LPPS	Low-pressure plasma spraying
EDM	Electrical discharge machining
VPS	Vacuum plasma spray
